# Higher‐level competence: Results from the Integrated Longitudinal Studies on Aging in Japan (ILSA‐J) on the shape of associations with impaired physical and cognitive functions

**DOI:** 10.1111/ggi.14839

**Published:** 2024-02-28

**Authors:** Takumi Abe, Yoshinori Fujiwara, Akihiko Kitamura, Yu Nofuji, Yukiko Nishita, Hyuma Makizako, Seungwon Jeong, Masanori Iwasaki, Minoru Yamada, Narumi Kojima, Katsuya Iijima, Shuichi Obuchi, Ken Shinmura, Rei Otsuka, Takao Suzuki

**Affiliations:** ^1^ Research Team for Social Participation and Healthy Aging, Tokyo Metropolitan Institute for Geriatrics and Gerontology Tokyo Japan; ^2^ Health Town Development Science Center, Yao City Public Health Center Osaka Japan; ^3^ Department of Epidemiology of Aging National Center for Geriatrics and Gerontology Obu Japan; ^4^ Department of Physical Therapy Faculty of Medicine, School of Health Sciences, Kagoshima University Kagoshima Japan; ^5^ Department of Community Welfare Niimi University Okayama Japan; ^6^ Department of Social Science National Center for Geriatrics and Gerontology Obu Japan; ^7^ Department of Preventive Dentistry Faculty of Dental Medicine and Graduate School of Dental Medicine, Hokkaido University Sapporo Japan; ^8^ Graduate School of Comprehensive Human Sciences, University of Tsukuba Tokyo Japan; ^9^ Institute of Gerontology, The University of Tokyo Tokyo Japan; ^10^ Institute for Future Initiatives, The University of Tokyo Tokyo Japan; ^11^ Department of General Internal Medicine Hyogo Medical University School of Medicine Nishinomiya Hyogo Japan; ^12^ National Center for Geriatrics and Gerontology Obu Japan; ^13^ Institute of Gerontology, J. F. Oberlin University Tokyo Japan

**Keywords:** functional capacity, gait speed, grip strength, Japan Science and Technology Agency Index of Competence, Mini‐Mental State Examination

## Abstract

**Aim:**

This study aimed to examine the relationships between levels of competence and impaired physical and cognitive functions in older adults.

**Methods:**

We used a data set of the Integrated Longitudinal Studies on Aging in Japan for 2017 including 5475 community‐dwelling older adults. Levels of competence were assessed using the Japan Science and Technology Agency Index of Competence (JST‐IC). Grip strength (low grip strength: <28 kg for men and <18 kg for women) and gait speed (slow gait speed: <1.0 m/s for both sexes) were evaluated as physical function measurements, and the Mini‐Mental State Examination (cognitive decline: <24 on the Mini‐Mental State Examination) was used to assess cognitive function.

**Results:**

The JST‐IC had areas under the curve estimated from receiver operating characteristic analysis ranging from 0.65 to 0.73 for detecting low function as assessed by these tests. Restricted cubic spline curves showed that the shape of the association between the JST‐IC and impaired function depended on sex and the test used. The comparison between perfect and imperfect JST‐IC scores showed significant differences in the prevalence of low grip strength in both sexes, slow gait speed in women, and cognitive decline in men.

**Conclusions:**

It may be insufficient to identify those with impaired physical or cognitive function using the JST‐IC. The shape of the association with the JST‐IC varies across their measurements. Our findings can help interpret JST‐IC scores in the context of low physical and cognitive functions. **Geriatr Gerontol Int 2024; 24: 352–358**.

## Graphical abstract

Using data from the Integrated Longitudinal Studies on Aging in Japan (*n* = 5475), this study found that the shape of the association between the Japan Science and Technology Agency Index of Competence and impaired physical and cognitive functions depends on the outcome measures (low grip strength, slow gait speed, and cognitive decline).

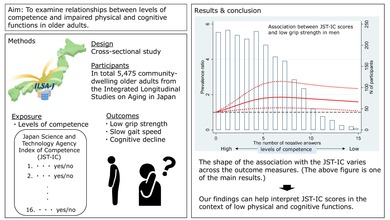

## Introduction

Higher levels of competence, which indicate the ability for independent and active living, have been a longstanding research topic.^1^, ^2^, ^3^, ^4^, ^5^ More than 50 years ago, Lowton proposed a model describing the competence of older adults,^1^ including seven sublevels (e.g. instrumental self‐maintenance and social role). Based on Lowton's model, the Tokyo Metropolitan Institute of Gerontology Index of Competence was developed in 1987 as a tool to assess the competence of older adults.^2^, ^3^ Afterwards, the Japan Science and Technology Agency Index of Competence (JST‐IC) with four subscales was developed in 2015 to assess competence adapting to changes in lifestyle (e.g., the prevalence of mobile phones and access to a large amount of information).^4^, ^5^ It may be a more suitable measurement than the Tokyo Metropolitan Institute of Gerontology Index of Competence for assessing modern life abilities.

The association of the level of competence with physical and cognitive functions is an important focus, as its assessment could be used as a surrogate for those functions when not conducting objective measurements (e.g. mail survey). A fundamental study on the JST‐IC has found that the JST‐IC score is correlated with self‐reported physical function.^5^ Another study has reported associations between the JST‐IC score and objective physical and cognitive functions.^6^ While these findings support that higher JST‐IC scores correspond to higher levels of physical and cognitive functions, there is no indication of how the JST‐IC score is associated with low levels of these functions as defined by the established cutoff points. Grip strength and gait speed are representative physical performance tests; both are used to define physical frailty and sarcopenia.^7^, ^8^ The Mini‐Mental State Examination (MMSE) is one of the most commonly used tests to assess cognitive function.^9^ Cutoff points have been established for these physical performance and cognitive tests to assess health status,^7^, ^8^ suggesting that those thresholds are likely risk indicators for adverse health outcomes.

This study aimed to examine the relationships of JST‐IC scores with low levels of physical and cognitive functions in community‐dwelling older adults. In particular, this study focused on the following objectives: (i) to determine the extent to which JST‐IC scores differentiate between individuals with and without those functional impairments; (ii) to describe the shape of the associations between JST‐IC scores and those functional impairments; and (iii) to explore the associations between the subscales of the JST‐IC and those functional impairments, which can provide insights into the specific nature of each subscale.

## Methods

### Participants

Data from the Integrated Longitudinal Studies on Aging in Japan (ILSA‐J) were used for this cross‐sectional study. The ILSA‐J includes 16 community‐based cohort studies conducted in Japan (as of 2023),^10^ and its outline has been described elsewhere.^11^, ^12^ Briefly, data obtained in 2007, 2012, and 2017 were integrated (a difference of ±1 year was accepted for individual data); moreover, the number of cohort studies that provided their data with the ILSA‐J has been increasing since the ILSA‐J project started in 2017. The inclusion criteria and survey system (e.g. who instructs physical and cognitive function tests and how they are conducted) depended on each cohort, but the ILSA‐J data set includes data from community‐dwelling older adults aged ≥65 years. This study used the data set for 2017 that includes the data on the JST‐IC. We extracted data from participants in a cohort study that included measurements of grip strength, gait speed, and MMSE and JST‐IC scores, as described in the next section. Since one study did not obtain information about living arrangements from most participants (79%), they were considered an ineligible sample. Consequently, this study included 5527 participants from seven cohort studies. This study was approved by the Committee of Ethics of Human Research at the National Center for Geriatrics and Gerontology (No. 1607).

### Physical performance measures

#### Grip strength

We used a Smedley dynamometer to measure maximum grip strength. Two trials were conducted, and the highest value was adopted.

#### Gait speed

We conducted a 5‐m walk test. Participants walked an 11‐m path including acceleration and deceleration phases (3 m each) at their usual pace more than one time. The number of trials differed by cohort. The best score was recorded when multiple trials were performed.

### Cognitive function measure

We used the MMSE to assess cognitive function.^9^ The range of the MMSE score is 0–30, and a high score indicates better cognitive function.

### JST‐IC

The JST‐IC is composed of 16 yes/no questions with four subscales: technology use, information practice, life management, and social engagement. A previous study described the details of the questions, showing the validity of the JST‐IC as an assessment tool for higher‐level competence among community‐dwelling older adults.^5^ The reliability of the JST‐IC was confirmed: The alpha coefficient was 0.86 for the all items and 0.65–0.81 for its subscales.^13^ One point was given for positive responses, with high scores indicating a high level of competence (score range, 0–16).

### Statistical analysis

First, we used receiver operating characteristic analysis to examine the discriminative ability of the JST‐IC in identifying those with low physical and cognitive functions. In this analysis, we calculated the area under the curve (AUC), sensitivity, and specificity based on a cutoff point determined using the Youden index (sensitivity + specificity − 1). The cutoff points for low grip strength and slow gait speed were based on the established criteria for frailty and sarcopenia.^7^, ^8^ Specifically, low grip strength was defined as <28 kg for men and <18 kg for women, and slow gait speed was defined as <1.0 m/s for both sexes, referring to the cutoff points used to define physical frailty and sarcopenia.^7^, ^8^ Cognitive decline was defined as an MMSE score <24.^14^

Second, we fitted a restricted cubic spline to estimate the shape of the associations between the JST‐IC and low physical and cognitive functions. The number of knots (3–5) for the restricted cubic spline was determined on the basis of the Akaike information criterion and visual determination. In this context, we run mixed‐effect modified Poisson regression models. The outcome was any one of the following: low grip strength, slow gait speed, or cognitive decline. We used the number of negative answers in the JST‐IC as an exposure variable to make the figures visually understandable. We further collected general variables that were likely to be obtained in each cohort when creating the ILSA‐J data set. Specifically, age, education, body mass index, living arrangement (living alone), and medical histories of diabetes, heart disease, and stroke were included in the models as covariates. The selection of these variables was in accordance with a knowledge‐based assumption.^15^ This approach was reasonable, as a similar covariate selection was made previously.^16^ Furthermore, two dummy variables were added to the models. One was “initial participation,” indicating whether the individual data provided by each cohort for the ILSA‐J database for 2017 were obtained from those who previously attended their surveys or not. This dummy variable was used to adjust for the retest effects. The other was “cohort study” to distinguish between the cohort studies. This variable was included as a random effect in the models. The models were mutually adjusted for grip strength, gait speed, and the MMSE score (e.g. when the dependent variable was grip strength, gait speed and the MMSE score were included in the model as covariates.).

Third, we examined the relationships between the JST‐IC score and low physical and cognitive functions using mixed‐effect modified Poisson regression models. The exposure and covariates were the same as those mentioned above, while exposure was a subscale of the JST‐IC.

All the analyses were conducted for each sex by using Stata version 17. The level of statistical significance was set at 0.05.

## Results

A total of 52 individuals with missing data were excluded, leaving 5475 participants for the analysis (Table S1). Table 1 shows the characteristics of the participants. Of the analytical sample, 63% were women, and 70% of the participants provided their data on their first participation in each cohort study.

**Table 1:** Characteristics of study participants

	Men (*N* = 2021)	Women (*N* = 3454)
	Mean ± SD, *n* (%), or median (Q1, Q3)	Mean ± SD, *n* (%), or median (Q1, Q3)
Age, years	75.5 ± 6.2	74.3 ± 5.6
Education		
≤9 years	361 (17.9)	712 (20.6)
BMI		
<18.5 kg/m^2^	113 (5.6)	313 (9.1)
18.5–24.9 kg/m^2^	1378 (68.2)	2436 (70.5)
≥25.0 kg/m^2^	530 (26.2)	705 (20.4)
Living alone	311 (15.4)	1013 (29.3)
Diabetes	329 (16.3)	295 (8.5)
Heart disease	329 (16.3)	358 (10.4)
Stroke	144 (7.1)	126 (3.7)
Physical performance		
Grip strength, kg	33.9 ± 6.6	21.7 ± 4.3
Low grip strength	355 (17.6)	556 (16.1)
Gait speed, m/s	1.31 ± 0.25	1.34 ± 0.26
Slow gait speed	206 (10.2)	303 (8.8)
Cognitive function		
MMSE score	29 (27, 30)	29 (27, 30)
Cognitive decline	94 (4.7)	116 (3.4)
JST‐IC score		
Total, points	12 (9, 14)	12 (9, 14)
Technology usage, points	4 (3, 4)	4 (2, 4)
Information practice, points	4 (3, 4)	4 (3, 4)
Life management, points	3 (2, 4)	3 (3, 4)
Social engagement, points	2 (1, 4)	1 (0, 3)

BMI, body mass index; JST‐IC, Japan Science and Technology Agency Index of Competence; MMSE, Mini‐Mental State Examination.

The cutoff point was 12 of 13 for all outcomes in men, while it depended on the outcomes in women (Table 2). The AUC ranged from 0.65 to 0.73 in men and from 0.65 to 0.72 in women.

**Table 2:** Cutoff points (95% CI), AUC, sensitivity and specificity when identifying low physical and cognitive functions using the JST‐IC

	Low grip strength	Slow gait speed	Cognitive decline
Men			
Cutoff point	12/13	12/13	12/13
AUC (95% CI)	0.65 (0.62–0.68)	0.67 (0.64–0.71)	0.73 (0.67–0.79)
Sensitivity (%)	74.7	77.7	80.9
Specificity (%)	49.0	47.4	46.1
Women			
Cutoff point	10/11	11/12	9/10
AUC (95% CI)	0.65 (0.62–0.67)	0.72 (0.69–0.75)	0.71 (0.66–0.77)
Sensitivity (%)	54.7	75.9	57.8
Specificity (%)	67.5	56.0	75.0

AUC, area under the curve; CI, confidence interval; JST‐IC, Japan Science and Technology Agency Index of Competence.

**Figure 1:**
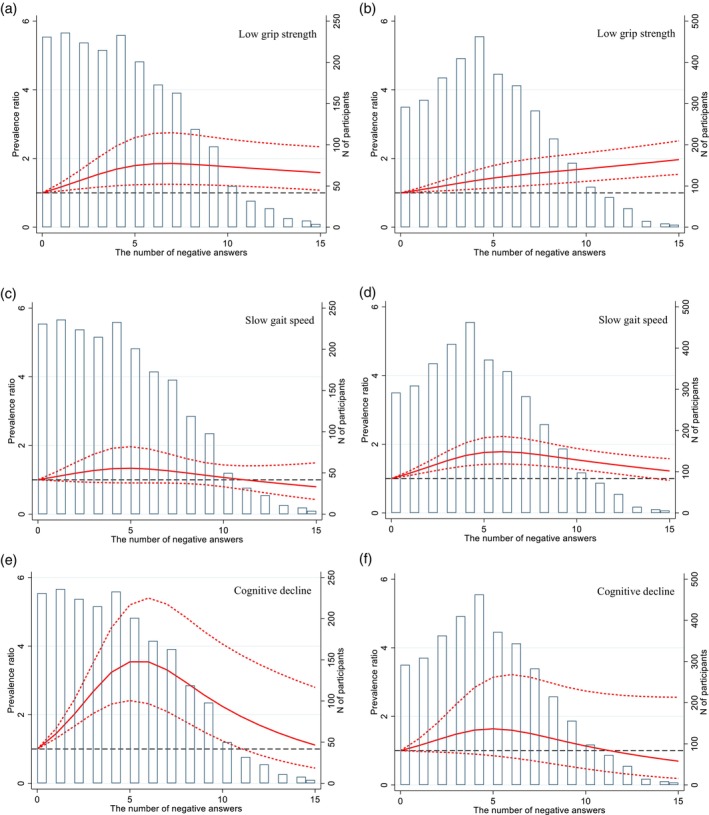
(a) Association between JST‐IC scores and low grip strength in men. (b) Association between JST‐IC scores and low grip strength in women. (c) Association between JST‐IC scores and slow gait speed in men. (d) Association between JST‐IC scores and slow gait speed in women. (e) Association between JST‐IC scores and cognitive decline in men. (f) Association between JST‐IC scores and cognitive decline in women. Solid lines and dashed lines mean prevalence ratio and 95% confidence interval, respectively. Low grip strength was defined as <28 kg for men and <18 kg for women. Slow gait speed was defined as <1.0 m/s for both sexes. Cognitive decline was defined as Mini‐Mental State Examination <24 points. Models adjusted for age, education, body mass index, living arrangement (living alone), diabetes, heart disease, stroke, and dummy variables indicating participants initially participated in the survey whose data were provided with the ILSA‐J data set for 2017 and a cohort each participant attended. Grip strength, gait speed, and the Mini‐Mental State Examination score were mutually adjusted. ILSA‐J, Integrated Longitudinal Studies on Aging in Japan; JST‐IC, Japan Science and Technology Agency Index of Competence.

Figure 1a–f shows the restricted cubic spline curves with three knots. Compared to the full score, the other scores showed a significantly higher prevalence ratio (PR) of low grip strength in both sexes (Figure 1a,b). In men, 9 points on the JST‐IC showed the highest PR (1.86 [95% confidence interval (CI), 1.25–2.75]; Figure 1a), whereas in women, as the JST‐IC score decreases, the PR increases (Figure 1b). There was no significant difference in the PRs of slow gait speed between full and the points of other JST‐IC scores in men (Figure 1c), but not full JST‐IC scores, except for 1 point, showed a significantly higher PR in women (10 points on the JST‐IC had the highest PR: 1.78 [95% CI, 1.43–2.23]; Figure 1d). Scores ranging from 5 to 15 points on the JST‐IC showed a higher PR of cognitive decline in men (Figure 1e), while no full JST‐IC scores did not have a significantly higher PR in women (Figure 1f).

The relationships between the JST‐IC subcomponent scores and low levels of physical and cognitive functions are shown in Table 3 for men and Table 4 for women. In most cases, the relationship depended on sex. A common trend in both sexes was that those who scored 0–3 points on technology use showed a high PR of low grip strength compared to those who scored 4 points.

**Table 3:** PR of low physical and cognitive function according to the JST‐IC subcomponent scores in men

	*n*	Low grip strength		Slow gait speed		Cognitive decline	
		*n* (%)	PR (95% CI)	*n* (%)	PR (95% CI)	*n* (%)	PR (95% CI)
Technology usage							
4 points	1265	148 (11.7)	Ref.	85 (6.7)	Ref.	24 (1.9)	Ref.
3 points	371	81 (21.8)	1.24 (1.03–1.49)*	54 (14.6)	1.10 (0.85–1.42)	19 (5.1)	0.96 (0.55–1.68)
2 points	209	52 (24.9)	1.22 (1.07–1.38)**	28 (13.4)	1.25 (0.84–1.88)	18 (8.6)	1.76 (1.10–2.83)*
0–1 point	176	74 (42.0)	1.28 (1.06–1.55)*	39 (22.2)	0.83 (0.62–1.13)	33 (18.8)	1.53 (0.54–4.31)
Information practice							
4 points	1183	182 (15.4)	Ref.	101 (8.5)	Ref.	28 (2.4)	Ref.
3 points	480	93 (19.4)	1.04 (0.87–1.24)	44 (9.2)	0.82 (0.64–1.03)	29 (6.0)	0.97 (0.63–1.47)
2 points	252	50 (19.8)	1.00 (0.75–1.32)	40 (15.9)	0.81 (0.55–1.18)	20 (7.9)	1.51 (1.03–2.21)*
0–1 point	106	30 (28.3)	1.10 (0.80–1.50)	21 (19.8)	1.52 (0.77–2.97)	17 (16.0)	0.72 (0.28–1.80)
Life management							
4 points	819	116 (14.2)	Ref.	51 (6.2)	Ref.	22 (2.7)	Ref.
3 points	567	84 (14.8)	0.90 (0.69–1.16)	62 (10.9)	0.96 (0.79–1.18)	25 (4.4)	2.00 (1.53–2.60)***
2 points	401	86 (21.4)	1.17 (0.81–1.69)	56 (14.0)	1.04 (0.84–1.28)	25 (6.2)	1.40 (1.06–1.85)*
0–1 point	234	69 (29.5)	1.29 (1.01–1.64)	37 (15.8)	0.65 (0.47–0.91)*	22 (9.4)	0.51 (0.21–1.22)
Social engagement							
4 points	585	69 (11.8)	Ref.	31 (5.3)	Ref.	12 (2.1)	Ref.
3 points	319	41 (12.9)	0.98 (0.57–1.68)	20 (6.3)	1.15 (0.89–1.48)	12 (3.8)	2.03 (1.12–3.68)*
2 points	260	47 (18.1)	1.27 (0.76–2.13)	25 (9.6)	1.17 (0.84–1.65)	8 (3.1)	1.77 (1.16–2.72)**
0–1 point	857	198 (23.1)	1.24 (0.79–1.94)	130 (15.2)	1.20 (0.90–1.60)	62 (7.2)	1.61 (1.12–2.30)**

Models adjusted for age, education, body mass index, living arrangement (living alone), diabetes, heart disease, stroke, and dummy variables indicating participants initially participated in the survey whose data were provided with the ILSA‐J data set for 2017 and a cohort each participants attended. Grip strength, gait speed, and the Mini‐Mental State Examination score were mutually adjusted.

CI, confidence interval; ILSA‐J, Integrated Longitudinal Studies on Aging in Japan; JST‐IC, Japan Science and Technology Agency Index of Competence; PR, prevalence ratio.

* *P* < 0.05;

** *P* < 0.01;

*** *P* < 0.001.

**Table 4:** PR of low physical and cognitive function according to the JST‐IC subcomponent scores in women

	*n*	Low grip strength		Slow gait speed		Cognitive decline	
		*n* (%)	PR (95% CI)	*n* (%)	PR (95% CI)	*n* (%)	PR (95% CI)
Technology usage							
4 points	1794	181 (10.1)	Ref.	84 (4.7)	Ref.	27 (1.5)	Ref.
3 points	781	138 (17.7)	1.37 (1.21–1.55)***	69 (8.8)	1.16 (0.94–1.42)	15 (1.9)	1.02 (0.61–1.72)
2 points	468	106 (22.6)	1.37 (1.15–1.64)**	66 (14.1)	1.49 (1.23–1.79)***	28 (6.0)	1.96 (1.61–2.37)***
0–1 point	411	131 (31.9)	1.44 (1.11–1.86)**	84 (20.4)	0.81 (0.47–1.39)	46 (11.2)	1.04 (0.74–1.46)
Information practice							
4 points	2054	280 (13.6)	Ref.	127 (6.2)	Ref.	47 (2.3)	Ref.
3 points	803	152 (18.9)	1.14 (0.97–1.34)	92 (11.5)	1.21 (1.09–1.36)**	30 (3.7)	1.40 (1.02–1.91)*
2 points	418	78 (18.7)	1.04 (0.92–1.17)	53 (12.7)	1.13 (0.95–1.34)	22 (5.3)	0.99 (0.73–1.34)
0–1 point	179	46 (25.7)	1.22 (1.01–1.49)*	31 (17.3)	1.43 (1.01–2.02)*	17 (9.5)	0.57 (0.12–2.82)
Life management							
4 points	1723	190 (11.0)	Ref.	83 (4.8)	Ref.	43 (2.5)	Ref.
3 points	943	162 (17.2)	1.30 (1.17–1.45)***	72 (7.6)	1.15 (0.99–1.33)	29 (3.1)	1.15 (0.64–2.08)
2 points	501	119 (23.8)	1.34 (1.17–1.53)***	82 (16.4)	1.05 (0.83–1.33)	25 (5.0)	1.21 (0.66–2.21)
0–1 point	287	85 (29.6)	1.45 (1.35–1.57)***	66 (23.0)	1.01 (0.83–1.21)	19 (6.6)	0.87 (0.39–1.95)
Social engagement							
4 points	703	75 (10.7)	Ref.	28 (4.0)	Ref.	10 (1.4)	Ref.
3 points	478	76 (15.9)	1.25 (1.08–1.45)**	2 (5.9)	1.28 (0.84–1.95)	13 (2.7)	1.48 (0.91–2.39)
2 points	539	74 (13.7)	1.03 (0.72–1.47)	45 (8.3)	1.18 (0.85–1.64)	12 (2.2)	1.00 (0.6–1.69)
0–1 point	1734	331 (19.1)	1.22 (1.05–1.42)*	202 (11.6)	1.11 (0.88–1.39)	8 (4.7)	0.95 (0.61–1.47)

Models adjusted for age, education, body mass index, living arrangement (living alone), diabetes, heart disease, stroke, and dummy variables indicating participants initially participated in the survey whose data were provided with the ILSA‐J data set for 2017 and a cohort each participants attended. Grip strength, gait speed, and the Mini‐Mental State Examination score were mutually adjusted.

CI, confidence interval; ILSA‐J, Integrated Longitudinal Studies on Aging in Japan; JST‐IC, Japan Science and Technology Agency Index of Competence; PR, prevalence ratio.

* *P* < 0.05;

** *P* < 0.01;

*** *P* < 0.001.

## Discussion

This study focused on the relationships between the JST‐IC score and low physical and cognitive functions. We found that the JST‐IC has acceptable discriminative ability for slow gait speed in women and cognitive decline in both sexes but could not be useful in identifying those with low grip strength in both sexes and slow gait speed in men. The shape of the association between lower JST‐IC scores and low physical function and cognitive decline depended on the outcome and sex. To some extent, there were associations between the JST‐IC subscales and low physical and cognitive functions.

Examining a score on the JST‐IC to distinguish between good and poor function is an important topic.^13^ Given that 0.70 of the AUC is a threshold indicating an acceptable level of discrimination,^17^ it could be reasonable to conclude that there is a limitation in identifying those with lower levels of physical and cognitive functions using the JST‐IC. Since the JST‐IC does not include items that indirectly assess grip strength, gait speed, or cognitive function (e.g. Can you open a stuck jar? Can you cross a pedestrian crossing while the lights are green? Can you plan for and manage this?), it may not show a high AUC for physical and cognitive functions.

A linear association was assumed when examining the relationship between the JST‐IC and physical and cognitive functions,^5^, ^6^ while our findings suggest nonlinear associations between the JST‐IC and low physical and cognitive functions (Figure 1a–f). Visualizing how PRs change as the JST‐IC decreases could be helpful in understanding the meaning of a score from the perspective of associations with low functions. Although this study did not find significant associations between the JST‐IC and slow gait speed in men and cognitive decline in women, the spline curves we showed can help speculate that those with lower JST‐IC scores are likely to have low physical and cognitive functions defined using an operational threshold.^7^, ^8^, ^9^ Considering that the 95% CI became wider in the case of lower JST‐IC scores, the accuracy of estimation for such scores requires careful interpretation and further investigation. For instance, low scores of the JST‐IC (e.g. <9 points, which are equivalent to <25th percentile in both sexes) did not necessarily show the highest PR of low physical and cognitive functions, except low grip strength in women, which may indicate that some older adults have such scores resulting from other factors such as poor social function or mental health but not those functions.

Relationships between the JST‐IC subcomponent scores and impaired physical function and cognitive decline differed between sexes in some respects. Of them, the score of technology usage showed the same trend in association with impaired physical and cognitive functions in both sexes (2 points had the highest PR of slow gait speed in both sexes, although the *P*‐value was >0.05 in men). Items for technology usage ask about the ability to use devices, whereas those for other subscales include questions about not only ability but also what those engage in. Not being able to do it and not doing it is different,^18^ but unlike questions about ability, questions about what older adults do cannot distinguish between them (i.e. some older adults do not do it even if they have the ability to do it). Although we do not have data on the proportion of those who cannot do so due to the nature of the JST‐IC, it might differ by sex, which could lead to the sex differences in the association between the subscale scores, as well as the total score, and impaired physical and cognitive functions. A study on the development of the JST‐IC has found that each subcomponent may have an individual practical use. For example, information practice scores were better correlated with health literacy (*r* = 0.58) than the other scores (*r* = 0.36–0.51), and social engagement scores had a higher correlation with social network (*r* = 0.40) than the other scores (*r* = 0.24–0.36).^5^ Given these results, the subcomponent scores may work to assess specific functions. On the other hand, our results suggest that they may not be useful to identify older adults with impaired physical and cognitive functions, except the relationship between technology use and low grip strength.

One strength of this study is that we analyzed data including objectively assessed physical and cognitive functions from >5000 community‐dwelling older adults. Because the ILSA‐J data set comprises community‐based cohort studies in Japan, our findings can be generalized to community settings. However, we acknowledge some limitations to our study. Our results would be subject to some bias (e.g. healthy user bias and reporting bias) and insufficient adjustments (e.g. economic status and chronic pain) owing to the nature of the ILSA‐J data set, which could lead to underestimation or overestimation of the relationships between the JST‐IC and impaired physical and cognitive functions. Additionally, we needed to rely on the definitions of low physical and cognitive functions based on just two physical performance tests and one cognitive test. Examining the association between the JST‐IC score and low physical and cognitive functions based on other standardized tests will be a future topic, although the tests we used are commonly used in community settings to define physical frailty and sarcopenia.

In conclusion, it may be insufficient to identify older adults with low grip strength, slow gait speed, or cognitive decline using a specific cutoff point of the JST‐IC score. Lower JST‐IC scores indicated a higher probability of having that status. In this context, dividing the total JST‐IC scores into subcomponent scores may not be helpful. Evidence of the JST‐IC was accumulated from cross‐sectional studies. Therefore, conducting a longitudinal analysis would be conducive to further understand the interpretation of JST‐IC scores.

## Disclosure statement

The authors declare no conflict of interest.

## Supporting information


**Table S1.** Descriptive data on main variables in each cohort (median (Q1, Q3) or mean ± SD).

## Data Availability

The data that support the findings of this study are available on request from the corresponding author. The data are not publicly available due to privacy or ethical restrictions.
