# Supplementary material for: Higher‐level competence: Results from the Integrated Longitudinal Studies on Aging in Japan (ILSA‐J) on the shape of associations with impaired physical and cognitive functions
Source: Geriatr Gerontol Int. 2024 Feb 28;24(4):352–8. doi: 10.1111/ggi.14839 (PMC11503746; doi:10.1111/ggi.14839)
Supplement: Supplementary file 1 — Table S1. Descriptive data on main variables in each cohort (median (Q1, Q3) or mean ± SD). [file GGI-24-352-s001.docx]

Supplementary Table 1. Descriptive data on main variables in each cohort (Median (Q1, Q3) or Mean ± SD)

|  | Kusatsu Longitudinal Study on Aging and Health | Otassha 2017 cohort | Otassha 2011 cohort | Takashimadaira Cohort Study | Kashiwa Nutrition and Health Study | Maibara study | FESTA study |
| --- | --- | --- | --- | --- | --- | --- | --- |
| N (women, %)^a^ | 1,243 (58.8) | 1,035 (100) | 831 (57.2) | 1,248 (59.5) | 950 (47.3) | 1,260 (54.6) | 873 (67.6) |
| n (women, %)^b^ | 480 (60.4) | 979 (100) | 767 (57.1) | 1,173 (59.2) | 927 (47.3) | 1,024 (51.3) | 177 (67.2)^c^ |
| Analytical sample (women, %) | 477 (60.4) | 979 (100) | 750 (56.9) | 1,168 (59.2) | 913 (47.5) | 1,012 (51.1) | 176 (67.1) |
| JST-IC score, points |  |  |  |  |  |  |  |
| Men | 12 (10, 14) | N/A | 12 (10, 15) | 10 (8, 13) | 12 (9, 14) | 14 (11, 15) | 14 (11, 15) |
| Women | 11 (9, 13) | 12 (9, 13) | 13 (11, 15) | 11 (8, 12) | 12 (9, 13) | 13 (11, 15) | 14 (12, 15) |
| Grip strength, kg |  |  |  |  |  |  |  |
| Men | 33.1 ± 6.9 | N/A | 34.1 ± 7.4 | 32.7 ± 6.6 | 34.1 ± 5.6 | 35.0 ± 6.7 | 35.3 ± 7.4 |
| Women | 20.0 ± 4.5 | 21.5 ± 3.9 | 21.3 ± 4.5 | 21.9 ± 4.6 | 22.9 ± 4.1 | 22.3 ± 4.1 | 22.5 ± 4.3 |
| Gait speed, m/s |  |  |  |  |  |  |  |
| Men | 1.3 ± 0.2 | N/A | 1.4 ± 0.3 | 1.2 ± 0.2 | 1.4 ± 0.2 | 1.3 ± 0.2 | 1.4 ± 0.2 |
| Women | 1.3 ± 0.2 | 1.4 ± 0.3 | 1.4 ± 0.3 | 1.3 ± 0.2 | 1.4 ± 0.2 | 1.3 ± 0.3 | 1.4 ± 0.2 |
| MMSE, points |  |  |  |  |  |  |  |
| Men | 29 (28, 30) | N/A | 28 (27, 29) | 28 (26, 29) | 29 (28, 30) | 29 (27, 30) | 28 (27, 29) |
| Women | 29 (27, 30) | 29 (27, 30) | 29 (28, 30) | 28 (26, 29) | 29 (28, 30) | 29 (27, 30) | 29 (26, 30) |

FESTA: Frail elderly in the Sasayama-Tamba area; JST-IC: Japan Science and Technology Agency Index of Competence; MMSE: Mini-Mental State Examination

^a^ The number of participants included in each cohort study

^b^ The number of participants who had data on JST-IC, grip strength, gait speed, and MMSE

^c^ The number of participants who attended the survey after starting data collection about living arrangements

Note: Kusatsu Longitudinal Study on Aging and Health was conducted in Kusatsu town, Gunma prefecture; Otassha 2017 cohort, Otassha 2011 cohort, and Takashimadaira Cohort Study in Itabashi ward, Tokyo; Kashiwa Nutrition and Health Study in Kashiwa city, Chiba prefecture; Maibara study in Maibara city, Shiga prefecture; and FESTA study in Sasayama-Tamba area, Hyogo prefecture.
